# Factors Associated with Knowledge of Diabetes in Patients with Type 2 Diabetes Using the Diabetes Knowledge Test Validated with Rasch Analysis

**DOI:** 10.1371/journal.pone.0080593

**Published:** 2013-12-03

**Authors:** Eva K. Fenwick, Jing Xie, Gwyn Rees, Robert P. Finger, Ecosse L. Lamoureux

**Affiliations:** 1 Centre for Eye Research Australia, University of Melbourne, The Royal Victorian Eye and Ear Hospital, East Melbourne Victoria, Austrailia; 2 Department of Ophthalmology, University of Bonn, Bonn, Germany; 3 Singapore Eye Research Institute, Singapore National Eye Centre, Singapore, Singapore; 4 Duke, Graduate Medical School, Singapore, Singapore; Endocrine Research Center (Firouzgar), Institute of Endocrinology and Metabolism, Islamic Republic of Iran

## Abstract

**Objective:**

In patients with Type 2 diabetes, to determine the factors associated with diabetes knowledge, derived from Rasch analysis, and compare results with a traditional raw scoring method.

**Research Design & Methods:**

Participants in this cross-sectional study underwent a comprehensive clinical and biochemical assessment. Diabetes knowledge (main outcome) was assessed using the Diabetes Knowledge Test (DKT) which was psychometrically validated using Rasch analysis. The relationship between diabetes knowledge and risk factors identified during univariate analyses was examined using multivariable linear regression. The results using raw and Rasch-transformed methods were descriptively compared.

**Results:**

181 patients (mean age±standard deviation = 66.97±9.17 years; 113 (62%) male) were included. Using Rasch-derived DKT scores, those with greater education (β = 1.14; CI: 0.25,2.04, p = 0.013); had seen an ophthalmologist (β = 1.65; CI: 0.63,2.66, p = 0.002), and spoke English at home (β = 1.37; CI: 0.43,2.31, p = 0.005) had significantly better diabetes knowledge than those with less education, had not seen an ophthalmologist and spoke a language other than English, respectively. Patients who were members of the National Diabetes Service Scheme (NDSS) and had seen a diabetes educator also had better diabetes knowledge than their counterparts. Higher HbA1c level was independently associated with worse diabetes knowledge. Using raw measures, access to an ophthalmologist and NDSS membership were not independently associated with diabetes knowledge.

**Conclusions:**

Sociodemographic, clinical and service use factors were independently associated with diabetes knowledge based on both raw scores and Rasch-derived scores, which supports the implementation of targeted interventions to improve patients' knowledge. Choice of psychometric analytical method can affect study outcomes and should be considered during intervention development.

## Introduction

It is estimated that 366 million people worldwide have diabetes and its prevalence is expected to increase considerably over the coming decades [1]. Management of diabetes is complex and multi-faceted. Many patients struggle to cope with the high level of self-care required to achieve the recommended diabetes control goals [2]. One of the barriers to good diabetes control is lack of knowledge about optimal diabetes control goals and associated self-care activities. For example, better knowledge of diabetes has been associated with greater likelihood to perform self-care activities (e.g. following a diabetes diet, blood glucose self-measurement and regular exercise) [3], fewer perceived barriers to blood glucose monitoring [4], and better medication adherence and glycaemic control [5]. Similarly, greater understanding of diabetes medications has been associated with better glycaemic control [6]. Poor diabetes health literacy has also been independently associated with worse glycaemic control and higher rates of diabetic retinopathy, a chronic and potentially blinding eye condition [7].

Several factors have been associated with poor diabetes knowledge, including lower educational level, older age, lower income, shorter diabetes duration, and lack of English language fluency [4], [8]–[14]. Of these, lower education level has consistently emerged as an independent risk factor for limited diabetes knowledge. Attending a diabetes education course, having health insurance and home glucose monitoring have been associated with better diabetes knowledge [8], [12]–[14]. As diabetes knowledge affects decisions about diet, exercise, weight control, blood glucose monitoring, medication use and prevention and treatment of microvascular and macrovascular diabetes complications, the American Diabetes Association and other national agencies have advocated patient education programs for diabetes [15], [16]. These educational interventions for diabetes have consistently been shown to improve patients' knowledge about diabetes, quality of life, self-care activities, fasting glucose, HbA1c level, cholesterol, blood pressure and waist circumference [17]–[19].

However, few studies have assessed the association between access to healthcare resources and health information, and diabetes knowledge, in addition to sociodemographic and clinical variables. Moreover, no studies have used a diabetes knowledge questionnaire validated using modern psychometric theory, such as Rasch analysis. Rasch analysis has several well-recognised advantages over summary scoring using raw measures as it converts ordinal-level raw data into interval measures that demonstrate the essential features of measurement and allow parametric testing to be performed [20]–[22]. Importantly, measurement precision is dramatically improved using Rasch-validated instruments compared to the summed scores, with studies reporting up to 148% increase in precision [23], [24]. It is therefore possible that previous findings associated with diabetes knowledge have under- or over-estimated the role of key risk factors and outcomes.

The purpose of this study was first to determine the sociodemographic, clinical and resource-related factors associated with limited diabetes knowledge in a clinical sample of patients in Australia with Type 2 diabetes using a Rasch-validated diabetes knowledge questionnaire. Second, we compared the factors associated with diabetes knowledge using raw and Rasch-transformed data.

## Materials and Methods

### Study design and participants

The Diabetes Management Project (DMP) is a longitudinal study that aims to determine the barriers to optimal diabetes control in patients with Type 1 and Type 2 diabetes. This sub-study reports on cross-sectional data from the baseline phase of the DMP which was conducted from March 2009 to December 2010 in Melbourne, Australia [25]. In total, 609 participants were recruited from specialised eye clinics at the Royal Victorian Eye and Ear Hospital (RVEEH) and Diabetes Australia-Victoria. Participants were eligible for the study if they were aged 18 years or older, English-speaking, free of significant hearing and cognitive impairment and living independently. Participants underwent a comprehensive assessment which included clinical, biochemical and anthropometric measures, and interviewer administered questionnaires on lifestyle, psychosocial factors, diabetes, knowledge, and self-care activities. The DMP cohort had a mean age of 64.60 (±11.6) and 65.5% (n = 399) were male. The majority (n = 510, 83.7%) had Type 2 diabetes. Of the full DMP sample, a sub-sample (n = 181) participants with Type 2 diabetes answered the Adapted Michigan Diabetes Research and Training Centre Brief Diabetes Knowledge Test (henceforth DKT) and were included in this study. Few differences between the current sample and the larger DMP cohort with Type 2 diabetes were identified. The sample included in this study were more likely to have one or more comorbidity, have private health insurance, and be slightly older (p<0.05) (**Table S1**).

### Ethics Statement

Each participant provided written informed consent and ethical approval was obtained from the RVEEH Human Research and Ethics Committee (08/815H). The study adhered to the tenets of the Declaration of Helsinki.

### Assessment of diabetes knowledge

The DKT is a 23-item multiple choice test designed to assess knowledge of diet, exercise, blood glucose levels and testing and self-care activities [26]. Each item has three or four possible answers with only one correct answer. The DKT was developed by diabetologists, dieticians, nurses, educational specialists and psychologists with expertise in diabetes and has been shown to be valid and reliable for a variety of settings and patient populations [4], [26]. The first 14 items are designed for all adults with diabetes, while items 15–23 apply only to those using insulin. Given that some of our study sample was not taking insulin, we administered the first 14 items only (**Appendix S1**). Diabetes knowledge is scored by summing the number of correct responses and converting the raw score to percentage correct.

### Psychometric validation of the DKT

Rasch analysis was used to assess the psychometric properties of the DKT scale using the dichotomous model [27] with Winsteps software (version 3.75), Chicago, Illinois, USA [28]. The dichotomous model was used since the items are scored as correct or incorrect. Rasch analysis is a form of Item Response Theory, where the ordinal ratings of the questionnaire are transformed to estimates of interval measures that demonstrate the essential features of measurement [29]. Analysis with the Rasch model provides difficulty measures for each item and ability estimates for each patient located on the same measurement scale in log of the odds units, or logits. Rasch analysis also provides significant insight into the psychometric properties of the scale [30] via the following key parameters.

Scale precision was assessed using the Person Separation Index (PSI) and Person Reliability (PR) coefficient [31]. A PSI value of 2.0 and PR coefficient of 0.8 indicate that the scale can successfully distinguish three levels of person ability which is the minimum level for a satisfactory scale [22].

The Rasch model requires that a scale is unidimensional, or that it measures a single underlying latent trait. Unidimensionality is assessed using item ‘fit statistics’ and testing the assumption of local independence. Items with an infit mean square standardized residual (MNSQ) value of 0.7–1.3 are considered acceptable [20]. Values below 0.7 may indicate redundancy and values over 1.3 indicate an unacceptable level of “noise” in the responses. Outfit MNSQ values may also be considered; however they are more susceptible to outliers. In principle component analysis (PCA) of the residuals, the first factor should explain at least 50% of the variance and the first contrast of the residuals (i.e. the second dimension) should be <2.0 eigenvalues [32]. A value of >2.0 is considered greater than that observed in random data and may suggest another dimension.

How well item difficulty targets person ability is assessed through visual inspection of the person-item map and the difference between person and item mean logits. A difference of >1.0 logits indicates notable mis-targeting [33]. Finally, we assessed for Differential Item Functioning (DIF) which occurs when sample subgroups respond differently to an item despite having similar underlying ability. DIF for gender and age group (≤65 years vs. >65 year) were investigated in this study. A DIF contrast of >1.0 logits and a corresponding p-value of <0.05 for an item is notable and suggests interpretation of the item differs by group.

### Assessment of risk factors

The main study outcome was diabetes knowledge assessed by the DKT using Rasch-validated measures. Key covariates included age (years); gender; duration of diabetes (years); insulin use; presence of one or more comorbidities (yes/no); presence of diabetic complications other than DR (yes/no); education level; household income; marital status; main language spoken at home; HbA_1_c (%); fasting plasma glucose (mg/dL); HDL cholesterol (mg/dL); systolic and diastolic blood pressure (SBP and DBP) (mmHg); body mass index (BMI) (kg/m^2^); and smoking status (non-smoker (ref)/current/past smoker). Access to relevant health care services was assessed by the following questions: ‘Have you ever seen a(n)…endocrinologist/ophthalmologist/diabetes educator/dietician/podiatrist/another health professional/another service?’ (yes/no). Patients were also asked if they had private health insurance, were a member of the National Diabetes Service Scheme (NDSS) and/or Diabetes Australia-Victoria (yes/no), and if they had noticed any health messages about diabetes in the media in the last 12 months (yes/no).

Diabetes self-efficacy was assessed using the 8-item Self-efficacy for Diabetes scale (Stanford Patient Education Research Centre) [34]. Individuals respond to using a 10-point Likert scale ranging from 1 (not at all confident) to 10 (totally confident) to assess confidence in performing diabetes self-care activities. Barriers to diabetes control were assessed using the 24-item Barriers to Diabetes Control questionnaire developed by the DMP research team [25]. The items are grouped into four categories, namely ‘diabetes management barriers’, ‘system barriers’, ‘psychosocial’ and ‘self-efficacy’. Items are rated with 3- or 4-category rating scales. Both the Self-efficacy for Diabetes and the Barriers to Diabetes Control questionnaires were validated in this study using Rasch analysis.

### Statistical analyses

Descriptive statistics were computed for all variables. Normality of the variables was examined using boxplots, Kolmogorov-Smirnov and Shapiro-Wilks tests. The linearity of nominal and ordinal data were assessed using Chi-square based measures. Diabetes knowledge as measured by the DKT scale (both raw and Rasch-transformed measures) was the main outcome and was analysed as a continuous variable. Univariate linear regression analysis was used to examine the relationship between diabetes knowledge and a variety of demographic, clinical, and psychosocial variables.

The relationship between diabetes knowledge (using both raw and Rasch-transformed measures) and the risk factors identified during univariate analysis were examined using a multivariable linear regression model and the results using raw and Rasch-transformed measures were descriptively compared. A plot of the residuals compared with estimates was examined to determine if the assumptions of linearity and homoscedasticity were met. Due to the large number of variables and the relatively small sample size, we used four criteria for evaluating linear regression models: R^2^_ADJ_ (adjusted), Akaike's information criterion (AIC), Akaike's corrected information criterion (AICc), and Bayesian information criterion (BIC). Generally, higher variance explained by the model (R^2^_ADJ_) and lower AIC, AICc and BIC values indicate the best fitting model. We used the Stata program, “vselect” to perform variable selection after performing linear regression [35]. We specified the best subset option to determine the best subsets of each predictor size. The four abovementioned criteria were considered for each of these subsets. All statistical analyses were conducted with Stata version 12.1.0 (Stata Corp, College Station, TX). A two-tailed p-value<0.05 was considered statistically significant.

## Results

### Psychometric validation of the DKT questionnaire

The DKT data were fitted to the Rasch model and the main validation indices were explored. The precision of the scale was adequate (PSI = 2.01 and PR = 0.80), and there were no misfitting items and no evidence of multidimensionality (raw variance explained by measures 50.5% and unexplained variance in the first contrast 1.6 eigenvalues). However, item 3 ‘Which of the following is highest in fat?’ displayed substantial DIF for age (DIF contrast 3.28, p<0.05) indicating that this item was more difficult for those in the older age group. A closer examination of item 3 showed that it had a very high Outfit MNSQ score (9.90) and was also at the extreme end of the difficulty spectrum meaning that very few patients endorsed it. Consequently, item 3 was deleted which improved the remaining fit statistics.

### Relationship between diabetes knowledge and patients' sociodemographic and clinical characteristics

One hundred and eighty-one participants answered the DKT, (62% male; mean age±standard deviation [SD] = 66.97±9.17 years. Performance on the DKT was poor, with the mean ‘percentage correct’ raw score on the DKT only 61.7±17.2%.

Using the Rasch transformed scores, the hardest item for participants to endorse (after removal of item 3) was item 8 ‘Which should not be used to treat low blood glucose?’ (**Figure S1**). Diabetes knowledge (Rasch transformed scores) was significantly higher in those with higher income, higher education level, in those who spoke English as their main language at home and who were currently employed (all p<0.001, **Table 1**). Similarly, those who had accessed health-related services to help manage their diabetes, such as an ophthalmologist, diabetic educator, or another service like counseling or support groups, and those who were members of the NDSS had better diabetes knowledge (all p<0.05, **Table 1**). Older age was significantly associated with worse diabetes knowledge (p<0.001). Non-significant associations between covariates and diabetes knowledge are reported in **Table S2**. Associations with DKT raw scores are provided in **Table S3**.

**Table 1:** Significant associations between diabetes knowledge (Rasch transformed scores) and sociodemographic and clinical variables (n = 181).

				Diabetes Knowledge	
Categorical variables	Mean	SD	p-value	β§ (95 CI)Δ	p-value
Income			<0.001		<0.001
<$30,000	0.60	1.81		0	
≥$30,000	2.34	3.05		1.74 (0.86, 2.62)	
Education level			<0.001		<0.001
High school or lower	0.67	1.86		0	
14 years or more	2.53	3.13		1.86 (0.97, 2.75)	
Language spoken at home (English)			0.02		<0.001
No	0.01	1.07		0	
Yes	1.52	2.66		1.51 (0.81, 2.22)	
Currently employed			<0.001		<0.001
No	0.94	2.20		0	
Yes	2.67	3.09		1.73 (0.60, 2.86)	
Private health insurance			0.018		0.027
No	0.99	2.32		0	
Yes	1.96	2.71		0.98 (0.11, 1.84)	
Member of NDSS			<0.001		<0.001
No	0.43	1.43		0	
Yes	1.57	2.71		1.14 (0.53, 1.77)	
Have you seen an ophthalmologist?			0.007		0.007
No	0.47	1.12		0	
Yes	1.34	2.57		0.88 (0.25, 1.50)	
Have you seen a diabetes educator?			0.002		0.001
No	0.61	1.93		0	
Yes	1.77	2.72		1.16 (0.47, 1.84)	
Have you seen a podiatrist?			0.09		0.07
No	0.82	2.10		0	
Yes	1.48	2.62		0.66 (−0.05, 1.36)	
Have you noticed any health messages about diabetes in the media in the last 12 months?			0.11		0.08
No	0.72	2.08		0	
Yes	1.39	2.54		0.68 (−0.07, 1.42)	
Have you used another service for your diabetes? (e.g. counselling, support groups, etc.)			0.001		0.05
No	1.12	2.33		0	
Yes	3.09	3.53		1.97 (−0.002, 3.94)	

Continuous variables	Mean (SD)	β§	95% CI	p-value
Age (years)	66.97 (9.17)	−0.08	−0.11, −0.04	<0.001
DBP Diastolic blood pressure (mmHg)	76.25 (7.97)	0.04	−0.01, 0.09	0.09
HbA1c %; (mmol/mol)	7.5 (1.7); 58 (18.6)	−0.20	−0.44, 0.03	0.09
Diabetes self efficacy	0.55 (0.87)	0.49	−0.08, 1.05	0.09

Variables significant at p<0.10 included.

§ regression correlation coefficient.

Δ univariate linear regression coefficient of risk factors for diabetes knowledge.

CI = Confidence interval; DBP = Diastolic blood pressure; NDSS = National Diabetes Service Scheme; SD = Standard Deviation.

### Determinants of diabetes knowledge: using Rasch Analysis

Based on the four criteria for evaluating multivariable regression models, three best-fitting models were chosen (**Table 2**). Model 1 included HbA1c level, access to an ophthalmologist (y/n), education level, currently employed (y/n), and NDSS member; model 2 included these variables (excluding NDSS member) plus age, language spoken at home, income, and access to a diabetes educator; model 3 included these variables plus NDSS member. These models each explained around 30% of the variance of diabetes knowledge.

**Table 2:** Variable selection for the Diabetes Knowledge Test (Rasch-transformed scores) in multivariable linear regression.

Model	R^2^_adj_	AIC	AICc	BIC
1	0.26	505.47	827.22	**521.83**
2	**0.31**	**501.07**	**823.91**	525.61
3	**0.31**	501.48	824.77	528.75

R^2^_adj_ = similar to the R^2^ measure (the proportion of variation “explained” by the regression model) but is corrected for the number of independent variables in the model. Higher values for this criterion indicate better fitting models.

AIC = Akaike's information criterion; AICc = a bias-corrected version of AIC; BIC = Bayesian information criterion. Lower AIC, AICc and BIC indicate better fitting models.

Bolded values indicate the ‘best’ value for each criterion and these four models represent the best models among all models specified for the data at hand.

Consistently across the three models, higher HbA1c level (β = −0.29; CI: −0.51,−0.07, p = 0.011) was significantly associated with worse diabetes knowledge (**Table 3**). Similarly, those with higher education level (β = 1.14; CI: 0.25,2.04, p = 0.013), who had accessed an ophthalmologist (β = 1.65; CI: 0.63,2.66, p = 0.002), and who spoke English at home (β = 1.37; CI: 0.43,2.31, p = 0.005) had significantly better diabetes knowledge (**Table 3**) than those with lower education level, who had not accessed an ophthalmologist and who spoke a language other than English, respectively. Three additional variables were seen in Models 1 and 2 only. Patients who were currently employed and who were members of the NDSS had better diabetes knowledge than those who were not (Model 1). Those who had seen a diabetes educator also had significantly better diabetes knowledge than those who had not (Model 2, **Table 3**).

**Table 3:** Determinants of diabetes knowledge (Rasch-transformed scores) in multivariable linear regression models.

	Model 1		Model 2		Model 3	
Variables	β (95 CI)	p	β (95 CI)	p	β (95 CI)	p
HbA1c	−1.67 (−3.14, −0.22)	**0.024**	−1.86 (−3.37, −0.35)	**0.016**	−2.04 (−3.63, −0.45)	**0.013**
Education level		**<0.001**		**<0.001**		**<0.001**
High school or lower	0		0		0	
14 years or more	10.11 (4.54, 15.67)		10.10 (4.70, 15.51)		10.86 (5.02, 16.70)	
Currently employed		**0.002**		0.112		0.100
No	0		0		0	
Yes	10.65 (4.09, 17.21)		5.82 (−1.37, 13.01)		6.38 (−1.24, 14.00)	
Language spoken at home (English)				**0.019**		0.172
No			0		0	
Yes			10.80 (1.83, 19.77)		6.29 (−2.78, 15.36)	
Have you seen a diabetes educator?				**0.007**		**0.014**
No			0		0	
Yes			7.39 (2.02, 12.77)		6.92 (1.41, 12.43)	
Have you noticed any health messages about diabetes in the media in the last 12 months?*				0.098		0.181
No			0		0	
Yes			5.57 (−1.05, 12.20)		4.82 (−2.28, 11.92)	
Marital status*						0.089
Married					0	
Not married					5.29 (−0.82, 11.41)	
Age			−0.32 (−0.66, 0.02)	0.066	−0.34 (−0.69, 0.00)	0.051

CI = Confidence interval; Bolded values indicate significant results;

* Represents variables substantially different from the analyses using Rasch-transformed scores (**Table 3**).

Model 1 had the smallest Bayesian information criterion (BIC).

Model 2 had the smallest a bias-corrected version of AIC.

Model 3 had the largest adjusted proportion of variation “explained” by the regression model.

### Determinants of diabetes knowledge: using raw DKT scores

Some of the same independent risk factors for poor diabetes knowledge using a Rasch-transformed method also emerged using raw DKT scores (summed respondent scores), namely higher HbA1c, lower education level, not currently employed, not accessing a diabetes educator and speaking a language other than English at home (**Table 4**). However, there were also some important differences. Access to an ophthalmologist and membership to the NDSS were significant factors associated with better diabetes knowledge using Rasch-transformed but not raw DKT scores. Moreover, noticing a health message about diabetes and marital status were (non-significant. p<0.05) factors associated with diabetes knowledge using the raw but not the Rasch-transformed DKT scores (**Table 4**).

**Table 4:** Determinants of diabetes knowledge (raw scores) in multivariable linear regression models.

	Model 1		Model 2		Model 3	
Variables	β (95 CI)	p	β (95 CI)	p	β (95 CI)	p
HbA1c	−0.29 (−0.51, −0.07)	**0.011**	−0.36 (−0.58, −0.14)	**0.001**	−0.37 (−0.58, −0.15)	**0.001**
Education level		**<0.001**		**0.029**		
High school or lower	0		0		0	**0.030**
14 years or more	1.66 (0.79, 2.54)		1.16 (0.12, 2.20)		1.14 (0.11, 2.18)	
Have you seen an ophthalmologist?*		**0.002**		**0.010**		
No	0		0		0	**0.010**
Yes	1.65 (0.63, 2.66)		1.77 (0.44, 3.11)		1.78 (0.43, 3.13)	
Currently employed		**0.003**		0.168		0.132
No	0		0		0	
Yes	1.76 (0.59, 2.92)		1.03 (−0.44, 2.50)		1.15 (−0.35, 2.65)	
Member of the National Diabetes Service Scheme (NDSS)*		**<0.001**				0.149
No	0				0	
Yes	1.21 (0.54, 1.87)				0.58 (−0.21, 1.38)	
Language spoken at home (English)				**0.005**		**0.011**
No			0		0	
Yes			1.37 (0.43, 2.31)		1.24 (0.29, 2.19)	
Have you seen a diabetes educator?				**0.039**		0.195
No			0		0	
Yes			0.82 (0.04, 1.61)		0.57 (−0.30, 1.45)	
Income				0.075		0.089
<$30,000			0		0	
≥$30,000			0.92 (−0.09, 1.94)		0.90 (−0.14, 1.93)	
Age			−0.05 (−0.11, 0.004)	0.069	−0.05 (−0.57, −0.15)	0.088

CI = Confidence interval; Bolded values indicate significant results NDSS = National Diabetes Service Scheme; SD = Standard Deviation.

* Represents variables substantially different from the analyses using raw scores (**Table 4**).

Model 1 had the smallest Bayesian information criterion (BIC).

Model 2 had the smallest a bias-corrected version of AIC.

Model 3 had the largest adjusted proportion of variation “explained” by the regression model.

## Discussion

Diabetes knowledge was limited in our sample of patients with Type 2 diabetes, suggesting that interventions to improve understanding of the condition are needed. Lower HbA1c level, higher education level, speaking English at home, accessing an ophthalmologist and a diabetes educator, and NDSS membership were independently associated with better diabetes knowledge. These findings indicate that diabetes-specific education and access to care is likely to improve diabetes knowledge and outcomes. We found similarities but also important differences in independent determinants of poor diabetes knowledge when comparing raw with Rasch-transformed DKT scores. This demonstrates the differences in research findings obtained from the use of ordinal data compared to interval measures as derived from Rasch analysis, which are appropriate for use in parametric analysis. Although several studies have highlighted the advantages of Item Response Theory (i.e. Rasch analysis) over Classical Test Theory (i.e. summed raw scores) methods, further studies are needed to confirm our data. Nonetheless, our study highlights that the choice of psychometric analytical method can have an impact on study outcomes and therefore should be evidence-based to ensure findings can confidently inform interventions and clinical practice.

Unlike most studies assessing diabetes knowledge [9], [10], [13], [14], [36], we found that worse glycaemic control was independently associated with worse diabetes knowledge. We found only two studies to support our finding [6], [11]. The inconsistent link between glycaemic control and diabetes knowledge may be due to the different scales used to assess diabetes knowledge which may lack items tapping into self-care activities like metabolic control. Moreover, the use of raw as opposed to Rasch-transformed scores may have masked potential associations in previous studies. Cultural differences may also be important, as previous studies have included Chinese, Kuwaiti, Mexican, American and Australian populations. Finally, a discrepancy between knowledge and self-care behaviour is common, and it is possible that other factors like service use and demographics may be more important determinants of knowledge than clinical factors.

Our finding that higher level of education and access to an ophthalmologist and diabetes educator were associated with better diabetes knowledge is supported by several other studies [8]–[14]. In a recent study of Chinese adults with Type 2 diabetes, participants who had more education, visited traditional Chinese medicine doctors and ophthalmologists and attended diabetes educational programmes had better diabetes knowledge [14]. Similarly, Bruce and associates found that greater education, attendance at diabetes education programs and visits to dieticians were independently associated with greater diabetes knowledge in a large sample of Australian patients with Type 2 diabetes [9]. The same study also found that English language fluency was independently associated with better diabetes knowledge [9], which is similar to our study where speaking English as the main language at home was associated with better diabetes knowledge.

Another important finding of the current study was that NDSS membership was associated with better diabetes knowledge. The NDSS is an initiative of the Australian Government administered by Diabetes Australia. The NDSS delivers diabetes-related products at subsidised prices and provides information and support services to people with diabetes, such as online fact sheets about a variety of diabetes-related topics. To our knowledge, no previous studies have assessed the impact of these types of support services on diabetes knowledge. Future longitudinal studies are required to determine causality in this relationship, namely if those with greater diabetes knowledge are more likely to join the NDSS, or if joining the NDSS improves diabetes knowledge.

Unlike many previous studies [4], [9], [10], [12]–[14], we did not find an association between older age and worse diabetes knowledge. Additional determinants of poor diabetes knowledge, including lower income [10], [11], shorter duration of diabetes [4], [13] and being a current smoker [10] have been previously reported, but these were not significant in our study. Similarly, three studies have reported an association between family history of diabetes and higher diabetes knowledge scores [8], [10], [14] which was not assessed in our study.

Some factors associated with limited diabetes knowledge in our study are modifiable and could be addressed in targeted interventions. For example, educational programmes to improve diabetes knowledge in the areas of diet, exercise, blood glucose levels and testing, and self-care activities could be developed and evaluated in clinical and community settings. In addition, improving awareness of the importance of accessing health professionals and support services at a primary, tertiary and community level such as ophthalmologists, diabetes educators and the NDSS could be targeted. Educational programmes are advocated by the American Diabetes Association and Diabetes Australia and have been empirically shown to improve clinical, behavioural and patient-centred outcomes relating to diabetes and associated complications [17]–[19]. Tailoring and targeting diabetes educational programs to those who speak English as a second language may also help improve diabetes knowledge in culturally and linguistically diverse groups.

Our results highlight that GPs and ophthalmologists should be encouraged to provide referrals to diabetes educators for patients they suspect have poor diabetes knowledge, especially those with limited English language skills. Provision of information about diabetes to patients by ophthalmologists may be valuable even if this goes beyond their usual scope of practice. Enlisting the support of other providers, such as diabetes educators, dieticians, and social and case workers by primary care physicians has been recognised as an important step to optimising diabetes care [37]. Moreover, use of multidisciplinary resources at the clinic level to improve diabetes care, such as nurse and dietician educators, has been linked with improvements in HbA1c and cholesterol levels in patients with diabetes [38].

Our comparison of Rasch-transformed and raw DKT scores is novel and demonstrates key differences in study findings between these two approaches. Importantly, access to an ophthalmologist and membership of the NDSS were not independently associated with diabetes knowledge using raw summed scores. As such, this information would not inform the development of interventions to improve diabetes knowledge which could reduce their efficacy in improving patient outcomes. This demonstrates that choice of psychometric analytical method can affect study outcomes and therefore should be evidence-based. The limitations of Classical Test Theory and summary scoring have been well-described [20]–[22] and studies have reported increased precision when using Rasch-transformed compared to summary scoring [23], [24].

Strengths of this study include the large number of variables assessed and the use of robust statistical techniques to determine the best fitting models, which each explained approximately 30% of the variance in test performance. Our use of Rasch analysis to validate the DKT in our sample ensured that our results demonstrated the essential features of measurement and allowed us to explore the relative difficulty of each of the 14 DKT items. Limitations include the cross-sectional study design which means that causal associations cannot be determined. For example, the association between knowledge and ophthalmic consultations may suggest that ophthalmologists are providing general diabetes education within consultations, or that those with greater diabetes knowledge are more likely to seek and attend an ophthalmic consultation. That participants were primarily recruited from tertiary eye clinics may limit the generalisability of the study results. Further research is required in additional samples such as patients newly diagnosed with diabetes or managed within primary care settings. Some of the data were self-reported, such as access to diabetes health professionals, which may have been affected by recall bias.

In conclusion, we found that higher HbA1c level was independently associated with worse diabetes knowledge, while higher education level, speaking English at home, accessing an ophthalmologist and diabetes educator, and NDSS membership were independently associated with better diabetes knowledge. Interventions to improve diabetes knowledge which focus on improving understanding of diabetes control and awareness of health-related services are needed. The difference in study findings using raw compared to Rasch-transformed DKT scores suggests that choice of psychometric analytical method can impact on study outcomes and should be evidence-based.

## Supporting Information

Figure S1 **Person-item map for the DKT scale before removal of item 3.**
(DOCX)

Table S1 **Comparisons of sociodemographic and clinical variables between the wider DMP cohort and respondent sample in DKT (n = 510).**
(DOCX)

Table S2 **Non-significant associations between diabetes knowledge (Rasch transformed scores) and sociodemographic and clinical variables (n = 181).**
(DOCX)

Table S3 **Significant associations between diabetes knowledge (raw score) and sociodemographic and clinical variables (n = 181).**
(DOCX)

Appendix S1 **Adapted Michigan Diabetes Research & Training Center Brief Diabetes Knowledge Test.**
(DOCX)
