# Supplementary material for: Factors Associated with Knowledge of Diabetes in Patients with Type 2 Diabetes Using the Diabetes Knowledge Test Validated with Rasch Analysis
Source: PLoS One. 2013 Dec 3;8(12):e80593. doi: 10.1371/journal.pone.0080593 (PMC3848993; doi:10.1371/journal.pone.0080593)
Supplement: Figure S1 — Person-item map for the DKT scale before removal of item 3. (DOCX) [file pone.0080593.s001.docx]

MEASURE PERSON - MAP - ITEM

<more>|<rare>

6 +

| q3

|

|

|

.##### |

5 +

|T

|

|

T|

| q8

4 +

. |

|

|

.# |

. |

3 +

|

|

S|

|S

.#### |

2 +

| q7

|

|

|

.######## |

1 + q5

|

M|

########### |

. |

|

0 .####### +M

. | q2

|

.######## | q1

. | q13

### |

-1 # + q10 q4

. S| q6

#### | q11 q12

|

|

## |

-2 +

|

. |S

| q14

|

|

-3 . T+

| q9

|

|

|

|

-4 +

<less>|<frequent>

**Figure S1.** Person-item map for the DKT scale before removal of item 3. To the left of the dashed line are the participants, represented by # signifying 3 participants and . signifying 1 to 2 participants, and on the right are the items, denoted by their item number. Participants with higher ability and the most difficult items are near the top of the diagram, and participants with lower ability and items that can be performed with least difficulty are near the bottom. M, mean; S, 1SD from the mean; T, 2 SD from the mean.
