# Supplementary material for: Factors Associated with Knowledge of Diabetes in Patients with Type 2 Diabetes Using the Diabetes Knowledge Test Validated with Rasch Analysis
Source: PLoS One. 2013 Dec 3;8(12):e80593. doi: 10.1371/journal.pone.0080593 (PMC3848993; doi:10.1371/journal.pone.0080593)
Supplement: Table S1 — Comparisons of sociodemographic and clinical variables between the wider DMP cohort and respondent sample in DKT (n = 510). (DOCX) [file pone.0080593.s002.docx]

**Table S1.** Comparisons of sociodemographic and clinical variables between the wider DMP cohort and respondent sample in DKT (n=510)

| **Categorical variables** | **No DKT (n=329)** | **DKT (n=181)** | **p** |
| --- | --- | --- | --- |
|  | **N (%)** | **N (%)** |  |
| **Gender** |  |  |  |
| Female | 110 (33.4) | 68 (37.6) | 0.349 |
| Male | 213 (66.6) | 113 (62.4) |  |
| Income |  |  |  |
| <$30,000 | 221 (74.2) | 112 (67.1) | 0.104 |
| ≥$30,000 | 77 (25.8) | 55 (32.9) |  |
| Education level |  |  |  |
| High school or lower | 233 (72.8) | 120 (68.2) | 0.276 |
| 14 years or more | 87 (27.2) | 56 (31.8) |  |
| Smoking status |  |  |  |
| Non-smoker | 147 (45.4) | 82 (45.6) | 0.968 |
| current/past smoker | 177 (54.6) | 98 (54.4) |  |
| Language spoken at home (English) |  |  |  |
| No | 55 (20.0) | 17 (12.8) | 0.073 |
| Yes | 220 (80.0) | 116 (87.2) |  |
| Currently employed |  |  |  |
| No | 265 (81.0) | 149 (82.3) | 0.772 |
| Yes | 62 (19.0) | 32 (17.7) |  |
| Number of comorbidities† |  |  |  |
| 0 | 58 (17.6) | 15 (8.3) | **0.004** |
| ≥1 | 271 (82.4) | 166 (91.7) |  |
| Diabetic complications‡ |  |  |  |
| 0 | 220 (66.9) | 125 (69.1) | 0.613 |
| ≥ 1 | 109 (33.1) | 56 (30.9) |  |
| Vision impairment |  |  |  |
| No | 128 (42.1) | 86 (50.0) | 0.096 |
| Yes (>0.3 LogMAR) | 176 (57.9) | 86 (50.0) |  |
| Presence of diabetic retinopathy |  |  |  |
| no | 123 (38.1) | 77 (44.2) | 0.181 |
| Any diabetic retinopathy | 200 (61.9) | 97 (55.8) |  |
| Private health insurance |  |  |  |
| No | 273 (83.5) | 133 (73.5) | **0.007** |
| Yes | 54 (16.5) | 48 (26.5) |  |
| Member of NDSS |  |  |  |
| No | 77 (28.0) | 49 (27.4) | 0.884 |
| Yes | 198 (72.0) | 130 (72.6) |  |
| Have you seen a diabetes educator? |  |  |  |
| No | 105 (47.5) | 79 (43.9) | 0.469 |
| Yes | 116 (52.5) | 101 (56.1) |  |
| Have you seen a podiatrist? |  |  |  |
| No | 68 (30.9) | 64 (35.4) | 0.345 |
| Yes | 152 (69.1) | 117 (64.6) |  |
| Have you used another service for your diabetes? (e.g. counselling, support groups, etc.) |  |  |  |
| No | 289 (88.6) | 169 (93.4) | 0.09 |
| Yes | 37 (11.4) | 12 (6.6) |  |
| **Continuous variables** |  |  |  |
| Age | 65.01 (11.08) | 66.97 (9.17) | **0.041** |
| SBP (mmHg) | 140.26 (18.93) | 141.44 (19.24) | 0.507 |
| DBP (mmHg) | 76.16 (9.34) | 76.25 (7.97) | 0.915 |
| Duration of diabetes (years)^Δ^ | 13.0 (13.0) | 12.0 (14.3) | 0.642 |
| BMI (kg/m^2^) | 31.49 (6.45) | 30.61 (5.89) | 0.135 |
| HDL cholesterol (mg/dL) ^Δ^ | 1.25 (0.54) | 1.34 (0.47) | **0.019** |
| Fasting plasma glucose (mg/dL) | 7.70 (3.30) | 7.70 (3.60) | 0.742 |

CI=Confidence interval; DBP=Diastolic blood pressure; NDSS=National Diabetes Service Scheme; SD=Standard Deviation. IQR= interquartile range.

^Δ:^ Median (IQR)
