# Supplementary material for: Factors Associated with Knowledge of Diabetes in Patients with Type 2 Diabetes Using the Diabetes Knowledge Test Validated with Rasch Analysis
Source: PLoS One. 2013 Dec 3;8(12):e80593. doi: 10.1371/journal.pone.0080593 (PMC3848993; doi:10.1371/journal.pone.0080593)
Supplement: Table S2 — Non-significant associations between diabetes knowledge (Rasch transformed scores) and sociodemographic and clinical variables (n = 181). (DOCX) [file pone.0080593.s003.docx]

| **Table S2.** Non-significant associations between diabetes knowledge (Rasch transformed scores) and sociodemographic and clinical variables (n=181) | | | | | | |
| --- | --- | --- | --- | --- | --- | --- |
|  |  | | | | **Diabetes Knowledge** | |
| **Categorical variables** | **Mean** | **SD** | **p-value** | | **β^§^ (95 CI)^Δ^** | **p-value** |
| Gender |  |  |  | |  |  |
| Female | 1.57 | 2.83 | 0.18 | | 0 | 0.20 |
| Male | 1.05 | 2.21 |  |  | -0.51 (-1.31, 0.28) |  |
| Marital status |  |  |  | |  |  |
| Married/defacto | 1.08 | 2.44 | 0.16 | |  | 0.16 |
| Never married/Divorced/separated Widowed | 1.64 | 2.53 |  |  | 0.55 (-0.24, 1.36) |  |
| Who do you live with? |  |  |  | |  |  |
| Husband/wife/partner | 1.16 | 2.48 | 0.82 | | 0 | 0.70 |
| Nobody | 1.33 | 2.34 |  |  | 0.17 (-0.69, 1.03) |  |
| Others | 1.48 | 2.63 |  |  | 0.31 (-0.78, 1.40) |  |
| Smoking status |  |  |  | |  |  |
| Non-smoker | 1.50 | 2.61 | 0.22 | | 0 | 0.22 |
| Current/past smoker | 1.04 | 2.34 |  |  | -0.45 (-1.19, 0.28) |  |
| Member of DAV |  |  |  | |  |  |
| No | 0.97 | 2.30 | 0.20 | | 0 | 0.20 |
| Yes | 1.44 | 2.52 |  |  | 0.47 (-0.25, 1.19) |  |
| Have you seen an endocrinologist? |  |  |  | |  |  |
| No | 1.00 | 2.14 | 0.14 | | 0 | 0.15 |
| Yes | 1.55 | 2.82 |  |  | 0.54 (-0.21, 1.29) |  |
| Have you seen a dietician? |  |  |  | |  |  |
| No | 1.05 | 2.24 | 0.28 | | 0 | 0.28 |
| Yes | 1.44 | 2.67 |  |  | 0.40 (-0.33, 1.12) |  |
| Have you seen another health professional for your diabetes? (e.g. acupuncture, chiropractor, naturopath, hypnotherapist) |  |  |  | |  |  |
| No | 1.18 | 2.37 | 0.18 | | 0 | 0.33 |
| Yes | 2.23 | 3.66 |  |  | 1.04 (-1.07, 3.16) |  |
| DM medication |  |  |  | |  |  |
| No | 0.84 | 2.35 | 0.34 | | 0 | 0.34 |
| Yes | 1.25 | 2.40 |  |  | 0.42 (-0.43, 1.27) |  |
| Number of comorbidities† |  |  |  | |  |  |
| 0 | 2.28 | 3.05 | 0.09 | | 0 | 0.16 |
| ≥1 | 1.15 | 2.39 |  |  | -1.13 (-2.68, 0.43) |  |
| Diabetic complications‡ |  |  |  | |  |  |
| 0 | 1.25 | 2.46 | 0.97 | | 0 | 0.97 |
| ≥ 1 | 1.24 | 2.49 |  |  | -0.01 (-0.80, 0.77) |  |
| Vision impairment |  |  |  | |  |  |
| No | 1.46 | 2.59 | 0.29 | | 0 | 0.21 |
| Yes (>0.3 LogMAR) | 0.99 | 2.29 |  |  | -0.47 (-1.20, 0.26) |  |
| Presence of diabetic retinopathy |  |  |  | |  |  |
| No | 1.24 | 2.46 | 0.81 | | 0 | 0.81 |
| Any diabetic retinopathy | 1.34 | 2.53 |  |  | 0.09 (-0.66, 0.85) |  |
| Self-reported depression |  |  |  | |  |  |
| No | 1.17 | 2.44 | 0.44 | | 0 | 0.45 |
| Yes | 1.55 | 2.67 |  |  | 0.38 (-0.62, 1.38) |  |
| Self-reported anxiety |  |  |  | |  |  |
| No | 1.26 | 2.51 | 0.83 | | 0 | 0.82 |
| Yes | 1.15 | 2.36 |  |  | -0.12 (-1.13, 0.90) |  |
| **Continuous variables** | **Mean (SD)** | | **β^§^** | **95% CI** | | **p-value** |
| SBP Systolic blood pressure (mmHg) | 141.45 (19.24) | | -0.002 | -0.02, 0.02 | | 0.87 |
| Duration of diabetes (years) | 12.0 (14.3) | | -0.01 | -0.05, 0.03 | | 0.42 |
| BMI (kg/m^2^) | 29.71 (7.74) | | 0.004 | -0.08, 0.09 | | 0.92 |
| HDL cholesterol (mg/dL) | 1.33 (0.47) | | -0.37 | -1.14, 0.38 | | 0.33 |
| Fasting plasma glucose (mg/dL) | 7.7 (3.6) | | -0.05 | -0.13, 0.02 | | 0.16 |
| Barriers to diabetes control | 1.73 (1.68) | | 0.22 | -0.13, 0.56 | | 0.22 |
| Variables non-significant at p>0.10  † Includes: hypertension, heart attack/angina, irregular heartbeat, stroke, high cholesterol, asthma, anaemia, migraine, arthritis, osteoporosis  ‡ Includes: nephropathy, peripheral vascular disease, neuropathy  ^§^ regression correlation coefficient  *Δ* univariate linear regression coefficient of risk factors for diabetes knowledge  BMI=body mass index; CI=Confidence interval; DBP=Diastolic blood pressure; DAV=Diabetes Australia-Victoria; HDL=High density lipoprotein; NDSS=National Diabetes Service Scheme; SBP=Systolic blood pressure; SD=Standard Deviation. | | | | | | |
