# Supplementary material for: Factors Associated with Knowledge of Diabetes in Patients with Type 2 Diabetes Using the Diabetes Knowledge Test Validated with Rasch Analysis
Source: PLoS One. 2013 Dec 3;8(12):e80593. doi: 10.1371/journal.pone.0080593 (PMC3848993; doi:10.1371/journal.pone.0080593)
Supplement: Table S3 — Significant associations between diabetes knowledge (raw score) and sociodemographic and clinical variables (n = 181). (DOCX) [file pone.0080593.s004.docx]

| **Table S3.** Significant associations between diabetes knowledge (raw score) and sociodemographic and clinical variables (n=181) | | | | | |
| --- | --- | --- | --- | --- | --- |
|  |  | | | **Diabetes Knowledge** | |
| **Categorical variables** | **Mean** | **SD** | **p-value** | **β^§^ (95 CI)^Δ^** | **p-value** |
| Income |  |  |  |  |  |
| <$30,000 | 57.72 | 15.58 | <0.001 | 0 | <0.001 |
| ≥$30,000 | 68.24 | 18.05 |  | 10.51 (4.91, 16.12) |  |
| Education level |  |  |  |  |  |
| High school or lower | 57.96 | 16.05 | <0.001 | 0 | <0.001 |
| 14 years or more | 69.83 | 17.08 |  | 11.87 (6.53, 17.21) |  |
| Language spoken at home (English) |  |  |  |  |  |
| No | 51.88 | 15.17 | 0.003 | 0 | <0.001 |
| Yes | 63.71 | 17.41 |  | 11.82 (4.01, 19.62) |  |
| Currently employed |  |  |  |  |  |
| No | 59.56 | 16.87 | <0.001 | 0 | <0.001 |
| Yes | 71.81 | 15.02 |  | 12.25 (6.38, 18.11) |  |
| Private health insurance |  |  |  |  |  |
| No | 59.61 | 17.07 | 0.004 | 0 | 0.004 |
| Yes | 67.59 | 16.20 |  | 7.97 (2.53, 13.42) |  |
| Member of NDSS |  |  |  |  |  |
| No | 56.28 | 15.42 | 0.005 | 0 | <0.001 |
| Yes | 63.87 | 17.50 |  | 7.59 (2.30, 12.87) |  |
| Have you seen a diabetes educator? |  |  |  |  |  |
| No | 57.07 | 15.92 | 0.002 | 0 | 0.001 |
| Yes | 65.55 | 17.24 |  | 8.47 (3.58, 13.67) |  |
| Have you seen a podiatrist? |  |  |  |  |  |
| No | 58.87 | 15.88 | 0.09 | 0 | 0.087 |
| Yes | 63.29 | 17.70 |  | 4.42 (-0.65, 9.49) |  |
| Have you used another service for your diabetes? (e.g. counselling, support groups, etc.) |  |  |  |  |  |
| No | 60.96 | 16.99 | 0.016 | 0 | 0.016 |
| Yes | 72.54 | 16.52 |  | 11.59 (2.16, 21.01) |  |
| **Continuous variables** | **Mean (SD)** | | **β^§^** | **95% CI** | **p-value** |
| Age (years) | 66.97 (9.17) | | -0.52 | -0.76, -0.28 | <0.001 |
| DBP Diastolic blood pressure (mmHg) | 76.25 (7.96) | | 0.36 | 0.03, 0.69 | 0.034 |
| HbA1c %; (mmol/mol) | 7.50 (1.65) | | -1.47 | -3.07, 0.11 | 0.068 |
| Diabetes self-efficacy | 0.55 (0.87) | | 3.26 | -0.52, 7.03 | 0.09 |
| Variables significant at p<0.10 included  † Includes: hypertension, heart attack/angina, irregular heartbeat, stroke, high cholesterol, asthma, anaemia, migraine, arthritis, osteoporosis  ‡ Includes: nephropathy, peripheral vascular disease, neuropathy  ^§^ regression correlation coefficient  *Δ* univariate linear regression coefficient of risk factors for diabetes knowledge  CI=Confidence interval; DBP=Diastolic blood pressure; NDSS=National Diabetes Service Scheme; SD=Standard Deviation. | | | | | |
