# Supplementary material for: Factors Associated with Knowledge of Diabetes in Patients with Type 2 Diabetes Using the Diabetes Knowledge Test Validated with Rasch Analysis
Source: PLoS One. 2013 Dec 3;8(12):e80593. doi: 10.1371/journal.pone.0080593 (PMC3848993; doi:10.1371/journal.pone.0080593)
Supplement: Appendix S1 — Adapted Michigan Diabetes Research & Training Center Brief Diabetes Knowledge Test. (DOCX) [file pone.0080593.s005.docx]

**Appendix S1**: Adapted Michigan Diabetes Research & Training Center Brief Diabetes Knowledge Test

1. The diabetes diet is:

a. the way most Australian people eat

b. a healthy diet for most people

c. too high in carbohydrate for most people

d. too high in protein for most people

2. Which of the following is highest in carbohydrate?

a, Baked chicken

b. Swiss cheese

c. Baked potato

d. Peanut butter

3. Which of the following is highest in fat?

a. Low fat milk

b. Orange juice

c. Corn

d. Honey

4. “25%” reduced fat” means:

a. The product is healthy

b. The product is 25% lower in fat that the usual product

c. There is 25g of total fat per 100g of the product

d. Suitable for people with diabetes

5. Glycosylated hemoglobin (hemoglobin A1) is a test that is a measure of your average blood glucose level for the past:

a. day

b. week

c. 6-10 weeks

d. 6 months

6. Which is the best method for testing blood glucose?

a. Urine testing

b. Blood testing

c. Both are equally good

7. What effect does unsweetened fruit juice have on blood glucose?

a. Lowers it

b. Raises it

c. Has no effect

8. Which should not be used to treat low blood glucose?

a. 3 hard sweets or lollies

b. 1/2 cup orange juice

c. 1 cup diet soft drink

d. 1 cup skim milk

9. For a person in good control, what effect does exercise have on blood glucose?

a. Lowers it

b. Raises it

c. Has no effect

10. Infection is likely to cause:

a. an increase in blood glucose

b. a decrease in blood glucose

c. no change in blood glucose

11. The best way to take care of your feet is to:

a. look at and wash them each day

b. massage them with alcohol each day

c. soak them for one hour each day

d. buy shoes a size larger than usual

12. Eating foods lower in fat decreases your risk for:

a. nerve disease

b. kidney disease

c. heart disease

d. eye disease

13. Numbness and tingling may be symptoms of:

a. kidney disease

b. nerve disease

c. eye disease

d. liver disease

14. Which of the following is usually not associated with diabetes:

a. vision problems

b. kidney problems

c. nerve problems

d. lung problems
